# Psychedelics and neural plasticity

**DOI:** 10.1186/s12868-023-00809-0

**Published:** 2023-06-30

**Authors:** Rachael Sumner, Kacper Lukasiewicz

**Affiliations:** 1 University of Auckland, Auckland, New Zealand; 2 Medical University of Bialystok, Bialystok, Poland

## Abstract

When it comes to studying neural plasticity and psychedelics, the numerous and diverse neuroscientific fields converging on the topic provide unique insight into a complex picture. This editorial will describe the major ways in which the known effects of psychedelics on plasticity are being studied. We lay out strengths of different techniques and the major gaps and room for future research, particularly in the translation of pre-clinical studies to human research.

## Introduction

The term ‘psychedelic’ was first used to describe the ‘mind manifesting’ effects of a small group of drugs by Humphry Osmond in the 1950s. Psychedelics describes a class of compounds that alter consciousness, producing one or more of the following effects: sensory and body disturbance, hallucination, delusion, or dissociation. These effects are often associated with positive symptoms that go beyond or expand typical perception (such as mystical experiences, ego dissolution). It is generally agreed that classic psychedelics are those that have a principally serotonergic pharmacodynamic effect (such as lysergic acid diethylamide (LSD), psilocybin, N,N-dimethyltryptamine (DMT)); non-classics are those that do not, such as ketamine.

It has recently been discovered that psychedelic drugs have profound effects on neuroplasticity (i.e., the alteration of neuronal structure and function). Molecularly, psychedelics lead to rapid neuronal excitation, generation of new synapses, and changes in the dendritic arbor [1]. It is not yet known whether the plasticity-generating effects of psychedelics are mechanistically separable from the psychedelic experience. The importance of the pro-plasticity effects of these drugs led to the coining of the neologism *psychoplastogen* [2], with almost all current known psychoplastogens being either psychedelic or novel psychedelic derived/informed compounds.

The interest in psychedelics and plasticity has occurred with a contemporaneous growth of research into the potential health benefits of psychedelics generally, and the role of neuroplasticity in mental and neurological illness such as depression, Alzheimer’s, addiction, and schizophrenia, in particular (for example: [3]).

What lies ahead for psychedelics includes the potential for advances in our understanding of the neurophysiology of neuroplasticity in humans, as well as the clinical translation of novel treatments for neurological and psychiatric illness. However, critical questions on how to study psychedelics and neuroplasticity remain. For example, how do we best translate lab bench based findings and animal models of plasticity and illness to human clinical and basic neuroscience outcomes? What is the relationship between psychedelic induced changes to plasticity and changes to complex human behavior, emotion, and/or cognition? What are the implications for the development of medicines, design of treatments, and our wider understanding of psychedelics and the brain?

## Main text

Studies on animal models indicate that psychedelics promote neuroplasticity both *in vitro* and *in vivo* (reviewed in: [4]). Although there are multiple receptors targeted by these compounds resulting in a complex mechanism of action [5], classic psychedelics primarily activate the serotonin 2A (5-HT_2A_) receptor [6]. In a highly detailed study, Ly and colleagues [1] have shown that classical psychedelics (5-HT_2A_ receptor agonists), such as LSD, DMT, and 2,5-dimethoxy-4-iodoamphetamine (DOI) stimulate synaptogenesis and formation of new dendrites. These structural plasticity changes were accompanied by functional changes measured with electrophysiological responses of the neurons. By using a 5-HT_2A_ receptor antagonist, ketanserin, Ly and colleagues confirmed that this process depends on 5-HT_2A_ receptor activation. They also showed that downstream molecular signaling pathways, which regulate psychedelic-induced plasticity, are tropomyosin receptor kinase B (TrkB) and mammalian target of rapamycin (mTOR) pathways.

Studies using rodent models confirmed that 5-HT_2A_ receptor pathway activation by psychedelics leads to neuroplasticity *in vivo*, and such activation - even restricted only to the cortex - is sufficient to evoke behavior typical for psychedelic effects (in this model, the head-twitch response) [6]. Psilocybin-induced structural plasticity changes in the mouse prefrontal cortex were also long-lasting [7], suggesting that it could be the mechanism of psychedelic-related therapeutic effects in humans.

Animal studies provide the advantage of being able to provide direct evidence for molecular phenomena such as plasticity changes. There have been attempts to translate findings from animal models to human studies on psychedelic-induced therapeutic effects, but there are missing gaps in our knowledge. A good animal model relies on three criteria of validity: face validity, predictive validity and construct validity. However, head-twitch behavior, which is typically used in rodent psychedelic research, lacks face validity [8]. For example, interestingly, ketanserin is sufficient to abolish head-twitch responses, but not psilocybin-induced structural plasticity [7]. Moreover, Hesselgrave and colleagues showed that ketanserin pretreatment does not affect the antidepressant effects of psilocybin in mice [9]. These examples highlight the important missing gaps in our understanding between animal models of psychedelic actions and their therapeutic effects in humans.

While there are shortcomings in preclinical research, current human research into psychedelics and the mechanisms of plasticity lags behind the animal model work. Evidence for changes to the plasticity of neural circuitry in humans often necessitates scaling up the method of enquiry and thus the mechanism of plasticity being studied. Along these lines, macroscale plasticity changes can be indexed by changes in white and grey matter structure (measured with magnetic resonance imaging (MRI)). In addition, the function of local and network changes can be interrogated using functional MRI (fMRI), magnetoencephalography (MEG) and electroencephalography (EEG). Examples can be found in research into ketamine, where changes to neural plasticity have been reported as evidenced by changes to white matter tractography using structural MRI [10], increases in long-term-potentiation-driven modification of evoked potentials recorded with EEG, and excitation via neural oscillations recorded with MEG (reviewed in: [11]). Furthermore, blood sampling permits the exploration of proteins, mRNA, DNA and other markers specifically related to mechanisms of neural plasticity. The most typically studied is brain derived neurotrophic factor (BDNF) [4].

Broadly, use of the term ‘plasticity’ in human neuroscience research can simply refer to lability and capacity for change. Plasticity of behavior, personality and cognition may be reported both with and without neuroimaging. Though beyond the biomechanism focused founding definition of ‘psychoplastogen’ [2], presumably these complex changes may in part represent the end product of changes to plasticity. As a result, discussions on the synergy between the window of enhanced plasticity and psychotherapy are underway [12]. Entangled in this is an ongoing debate on the subjective effects of psychedelics and whether they are necessary to evoke therapeutic effects [13].

## Summary

Amongst considerable promise for psychedelics as future medicines, and in the broadening of our understanding of the brain, consciousness, cognition, and health, there is a wealth of knowledge still to be gained, and so many questions outstanding. This special Collection “Neural Plasticity and Psychedelics” in *BMC Neuroscience* is a timely opportunity to bring together different techniques and contexts of investigation on psychedelics and psychedelic-induced neural plasticity.

## Data Availability

N/A.

## Abbreviations

DOI: 2,5-dimethoxy-4-iodoamphetamine
BDNF: Brain derived neurotrophic factor
EEG: Electroencephalography
fMRI: Functional magnetic resonance imaging
LSD: Lysergic acid diethylamide
MRI: Magnetic resonance imaging
MEG: Magnetoencephalography
mTOR: Mammalian target of rapamycin
DMT: N,N-Dimethyltryptamine
5-HT_2A_: Serotonin 2A receptor
TrkB: Tropomyosin receptor kinase B
